# Stand-Alone and Combinatorial Effects of Plant-based Biostimulants on the Production and Leaf Quality of Perennial Wall Rocket

**DOI:** 10.3390/plants9070922

**Published:** 2020-07-21

**Authors:** Maria Giordano, Christophe El-Nakhel, Gianluca Caruso, Eugenio Cozzolino, Stefania De Pascale, Marios C. Kyriacou, Giuseppe Colla, Youssef Rouphael

**Affiliations:** 1 Department of Agricultural Sciences, University of Naples Federico II, 80055 Portici, Italy; maria.giordano@unina.it (M.G.); christophe.elnakhel@unina.it (C.E.-N.); gcaruso@unina.it (G.C.); depascal@unina.it (S.D.P.); 2 Council for Agricultural Research and Economics (CREA) – Research Center for Cereal and Industrial Crops, 81100 Caserta, Italy; eugenio.cozzolino@crea.gov.it; 3 Department of Vegetable Crops, Agricultural Research Institute, 1516 Nicosia, Cyprus; m.kyriacou@ari.gov.cy; 4 Department of Agriculture and Forest Sciences, University of Tuscia, 01100 Viterbo, Italy; giucolla@unitus.it

**Keywords:** *Diplotaxis tenuifolia* (L.) DC., plant biostimulant, vegetal protein hydrolysates, synergism, sustainable agriculture, mineral content, qualitative parameters

## Abstract

Modern agriculture is facing many difficulties due to a rapidly changing climate, and environmental damage from agricultural production. The commitment of scientists and farmers to increase environmentally sustainable agricultural practices is one way to help mitigate environmental impacts. Among these practices, the use of biostimulants could be beneficial for increasing fertilizer efficiency and reducing excessive use in agriculture, and as plant growth regulators capable of increasing both production volume and quality of crops. In our study, rocket plants were grown in a greenhouse and treated with two biostimulants (protein hydrolysates or tropical plant extract), either individually or combined, to assess the effect on yield, dry biomass, mineral content, qualitative parameters as well as on economic profitability of foliar biostimulant applications. Total yield and dry biomass of the plants treated with the three biostimulant combinations on average increased by 48.1% and 37.2% respectively compared to untreated plants, without significant differences between treatments. Biostimulant application increased the content of chlorophyll, K, Mg and Ca, compared to the untreated plants. Furthermore, a biostimulant synergistic effect was detected concerning the content of total ascorbic acid. Our results confirmed that the biostimulants are eco-friendly products, able to boost plant growth and product quality and thus increase growers’ profitability.

## 1. Introduction

Biostimulants are molecules and microorganisms that have been shown to have physiological and molecular effects on plants [1]. Their presence in agriculture is taking on an increasingly important role, as they can elicit specific agronomic responses, by enhancing chemical fertilizers effectiveness, increasing the growth and productivity of crops and improving the resistance of plants to constantly changing environmental conditions [1]. Defined by the scientists of the European Biostimulant Industry Council (EBIC), biostimulants are currently subjected to EU regulation, with the aim of facilitating their legalization in different European countries [1]. Biostimulants are distinguished from fertilizers due to their lower dose response and because they are not necessary for growth like fertilizer components [2]. They have been grouped into two main categories: microbial and nonmicrobial. Nonmicrobial biostimulants are represented by humic acids, protein hydrolysates and algae extracts. Microbial biostimulants are mycorrhizal fungi and plant growth-promoting rhizobacteria (PGPR), such as *Azospirillum*, *Azotobacter*, *Rhizobium* spp. The category of plant-derived protein hydrolysates are mixtures of free amino acids, oligo- and polypeptides, which are applied to leaves as a spray or directly into the soil in both greenhouse and field operations. They have shown positive effects on growth, productivity and tolerance to abiotic stress, with direct or indirect action [3,4]. They increase the metabolism of carbon (C) and the absorption of nitrogen (N) by directly stimulating the activity of enzymes, such as nitrate reductase (NR), nitrite reductase (NiR), glutamine synthetase (GS), glutamate synthase (GOGAT), citrate synthase, malate and isocitrate dehydrogenase [4]. Their indirect action is carried out by increasing plant roots in size and length [4]. Protein hydrolysates can be a source of energy and carbon for the microbial soil population, which can benefit plants as well [4,5]. Currently, protein hydrolysates, along with humic acids, cover 50% of the world biostimulant market, while seaweed extracts cover 37%. Knowledge of modalities and mechanisms of biostimulants action are still evolving. Dose trials and combination trials of different biostimulants have been used to establish their efficacy [6], but trials have not been able to identify physiological impacts of biostimulants. The so-called omic sciences (metabolomics, genomics, transcriptomics), together with phenotyping technologies, are seeking to identify interactions that can be established between different categories of biostimulants [7].

Combining two or more biostimulants can lead to additive, synergistic or antagonistic interactions, where the combined effect can be different from that obtained by each biostimulant individually. In fact, Colla et al. [3] showed that plants can respond differently to protein hydrolysates and plant extracts at different concentrations. An example is the mycoparasitic action of some *Trichoderma* spp. on fungal hyphae, which leads to a reduction in the absorption of phosphorus (P) in mycorrhized plants. In the additive interaction, each biostimulant maintains the same action as when used alone, and therefore each one of them adds its own action to the overall effect of the mixture. However, in the synergistic interaction, the overall effect of the mixture is greater than the sum of the effects of each biostimulant applied individually.

Limited scientific data are found in literature on the real interaction between biostimulants. Some examples illustrate that mixtures of protein hydrolysates, plant and seaweed extracts increase plant nutrient use efficiency and yield of tomato plants up to 11% [3], and co-inoculation with two different plant endophytic fungi (arbuscular mycorrhiza fungi and *Trichoderma*) increased the yield of lettuce plants 70% and zucchini 14% [4], resulting in a reduction of fertilizer application. Rouphael et al. [7], demonstrated that three different biostimulants improved yield performance and quality of greenhouse spinach plants, revealing different mechanisms of their action. Filippo-Herrera et al. [8], examined the effect of algae extracts in different concentrations on the germination and growth of mung bean plants. The seaweed extracts were rich in nutrients, such as amino acids. Some of the blends showed an increase in germination rate of up to 9%, an increase in average shoot length up to 21%, and an increase in root length of 36% as compared to the individual extracts. Plants’ fresh weight also increased by 32% and the dry weight by 45%, with some of the mixed extracts, as compared to the untreated control. However, some of the mixtures showed an antagonistic effect on germination.

Italy is the European leader of leafy greens production with about 15,000 ha in protected cultivation that are mainly produced in Campania region (southern Italy) as well as in Veneto and Lombardy (northern Italy). Over the past two decades, interest in perennial wall rocket (*Diplotaxis tenuifolia* (L.) D.C.), an important leafy vegetable from the Brassicaceae family, has been on the rise, driven by the growing interest in healthy diets [9,10]. Indeed, *Diplotaxis tenuifolia* is rich in minerals and phytochemical compounds, (i.e., carotenoids, flavonoids, glucosinolates, phenols and vitamin C) [11,12,13]. Under greenhouse conditions, perennial wall rocket is grown from autumn to spring, where three to five harvests can be made when the leaves have reached 10 to 15 cm in length, whereas a one to two harvests can be performed in the summer growing season [9]. Perennial wall rocket has spread in most agricultural areas, where an annual cultivation area of about 4000 hectares under greenhouse conditions has been reported [9]. To fulfill the high year-round demand and to improve perennial wall rocket yield to a level resulting in net economic benefits, the use of plant biostimulants could be a promising innovation for the realization of such a paradigm.

Soil fertility and microbial diversity, as well as animal and human health, are compromised by the excessive use of fertilizers and pesticides [5]. Very promising plant biostimulants are those based on protein hydrolysates and consisting of amino acids and small peptides, as well as those extracted from tropical plants containing not only amino acids and soluble peptides but also carbohydrates and vitamins. Understanding and optimizing the interactions between plant-based biostimulants could increase plant nutrient use efficiency, and reduce multiple abiotic stress, which can improve yield stability. Biostimulant mixtures with enhanced and specific actions can increase agricultural production, necessary for a growing world population, and to tackle this problem in a sustainable way.

The aim of this work was to evaluate the effect of two different plant-based biostimulants (legume-derived protein hydrolysates, and tropical plant extract), individually or in combination, on growth, nutritive qualities and economic profitability of perennial wall rocket (*Diplotaxis tenuifolia* (L.) D.C.) grown in a greenhouse, and to discern the kind of interaction between these two types of biostimulants on perennial wall rocket.

## 2. Results

### 2.1. Yield and Dry Biomass Production

Marketable yield and dry biomass were unaffected by the application of biostimulants and their combination in the first harvest (Table 1). Whereas, in the second harvest, both parameters increased on average by 47.1% and 46.9%, respectively, compared to the untreated control, with no significant difference between the three biostimulant treatments. In the third harvest, the yield increased on average by 95.2% when rocket was treated by the two biostimulants alone or in combination (Table 1). Noting that the dry biomass in protein hydrolysate (PH) + tropical plant extract (PE) treatment in the last two harvests was not significantly different from the other two biostimulants applied individually. The total yield and total dry biomass increased on average by 48.1% and 37.2%, respectively, compared to the untreated control plants, where no significant difference among biostimulants and their combination was noted. As biostimulant treatments did not have a significant effect on the crop’s yield and biomass, harvested at the first harvest. Physiological, mineral and qualitative analyses corresponding to this harvest were not assessed.

### 2.2. Soil Plant Analysis Development (SPAD) index and International Commission on Illumination (CIELAB) Color Parameters

SPAD index was significantly influenced by the biostimulant treatment in the second and third harvest (Table 2). Particularly, an average increase of 22.4% and 21.8% was recorded in the second and third harvest, respectively for wall rocket plants treated with biostimulants applied individually or combined (Table 2). As for the CIELAB color space parameters (L*, a* and b*), no significant difference was observed between treated and untreated control plants in the second as well as in the third harvest.

### 2.3. Leaf Mineral Composition

Biostimulants treatments (individually or combined) did not have a significant effect on total nitrogen, phosphorus and sodium content in rocket plants in any of the harvests (Table 3). Meanwhile, potassium content was greater in rocket plants treated with biostimulants compared to the untreated control, but was not significant: 11.5% on average in the second harvest, and 44.3% in the third harvest (Table 3). Calcium content increased by 31.7% (second harvest) and 29.3% (third harvest) when treated with PH, increased by 38.9% (second harvest) when treated with PE, and by 37.2% (second harvest) and 23.2% (third harvest) when the two biostimulants were combined together, compared to the control (Table 3). Magnesium content was not affected by biostimulants treatment in the second harvest, while an average increase of 37.9% was seen in the third harvest, compared to control plants, without any significant difference between the treatments (Table 3).

### 2.4. Qualitative Parameters

Biostimulants treatments did not have any significant effect on nitrate and phenolic content of perennial wall rocket in both harvests. While chlorophyll content was not statistically significant in the second harvest between treatments, it was significant in the third harvest when treated with PE, which increased by 22% and the combined treatment which increased by 33.3%, compared to the control (Table 4). Note that PH applied individually did not cause any significant increase. Total ascorbic acid content increased by 62.8% with PE application in the second harvest and 109.4% in the third harvest, compared to the control (Table 4). Combined treatment increased total ascorbic acid content by 115.0% in the second harvest and 143.6% in the third harvest, whereas PH treatment alone did not have any significant difference, compared to the control.

### 2.5. Principal Component Analysis (PCA)

**Figure 1:**
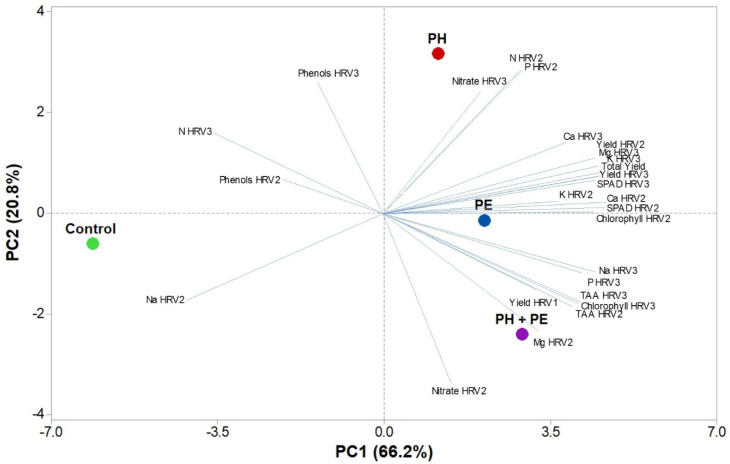
Principal component loading plot and scores of principal component analysis (PCA) of all agronomical and qualitative parameters of perennial wall rocket harvested (HRV) three times during the cultivation cycle, treated with either PE or PH or combined plant-based biostimulants (PH + PE).

The PCA of all agronomical and qualitative parameters of perennial wall rocket harvested three times during the cultivation cycle and treated with PH or PE individually or in combination, highlighted that the first three principal components (PCs) were related with eigenvalues higher than 1 and explained 100.0% of the total variance, with PC1, PC2 and PC3 resulting at 66.2%, 20.8% and 13.0% respectively (Table S1). The foliar application of plant-based biostimulants contributed to the separation of PC1 and PC2, as highlighted in the PCA output, revealing common trait variations among the plant biostimulant used. The effectiveness of PCA plotting plant biostimulant effects has been documented in several scientific papers dealing with the use of biostimulants on vegetable species [7,14,15]. This was observed in the ongoing greenhouse trial, since PCA scores in Figure 1 presented concerted knowledge on perennial wall rocket performance and nutritive value in relation to foliar biostimulants application. The first two PCs’ score plots separate biostimulant treatments into three groups (i.e., quadrants; Figure 1). The upper right quadrant of the positive side of PC1 consisted of the commercial plant biostimulant PH applied individually that delivered wall rocket leaves of high P and N (HRV2) contents (Figure 1). Interestingly, the combination of the two biostimulants (PH + PE) was located on the lower right quadrant of the positive side of PC1 representing perennial wall rocket with superior quality attributes (high total ascorbic acid). Finally, the lower left quadrant outlined the untreated control treatment which was characterized by the lowest quality attributes. Overall the loading plots and scores of our PCA may provide important information on the effect of foliar application of plant-based biostimulants alone or in combination on quality characteristics of greenhouse perennial wall rocket.

### 2.6. Partial Budget Analysis (PBA) of Biostimulant-Treated Wall Rocket Production

In the present greenhouse experiment, the added rocket fresh yields due to the foliar application of plant-based biostimulants compared to the untreated control were 8.6, 7.5 and 8.3 tons per hectare for PH, PE and their combination PH + PE, respectively (Table 1). Therefore, the beneficial effects associated with the foliar application of plant-based biostimulants involved the added gross returns on fresh perennial wall rocket values, which ranged between 9905 and 11354 Euro ha^−1^, with the highest added gross returns recorded in rocket plants treated with legume-derived protein hydrolysates (PH) (Table 5). Among the three added variable costs, biostimulant treatment was by far the main cost item (48% to 54% of total costs), followed by foliar spraying (26% to 28% of total costs) and finally the cost of machine harvest (20% to 24% of total costs) (Table 5). Moreover, the net returns of biostimulant treated to untreated plants were 3142, 9945, 8408 and 9812 for perennial wall rocket treated with PH, PE, and PH + PE, respectively (Table 5). Overall, our results indicated that the increased net economic benefit was associated with the significant improvement of fresh yield, in particular with PH and to a lesser extent with PH + PE, making it more profitable than untreated rocket production.

## 3. Discussion

In our work, following the application of plant-based biostimulants, both yield and dry biomass of rocket plants increased as compared to the control. Dry matter content in rocket plants is estimated to be around 90 g kg^−1^ [16], which is similar to our results (average 85 g kg^−1^). Such increase is supported by several works such as Caruso et al. [10] and Di Mola et al. [17], who both tested PH and PE on perennial wall rocket and baby rocket, respectively. Additionally, in the work of Colla et al. [3], the use of PH and PE positively influenced the total marketable yield of tomato plants grown in a greenhouse. The authors reported that some bioactive molecules, present in the two biostimulants (i.e., free amino acids, soluble peptides and vitamins), have an activity similar to auxin and gibberellin, also known as hormone-like activity. In the work of Carillo et al. [15], mobilization of N reserves (nitrate and amides) from roots to shoots was shown, with a consequent increase in leaf area, leaf dry matter and yield of spinach plants treated with PH also under suboptimal N rates. This is probably because the commercial PH Trainer^®^ contains an important amino acid such as tryptophan, which is a precursor of indole−3-acetic acid (IAA), responsible for the lengthening of shoots and roots. Other studies [18] reported an increase in the expression of the genes of glutamate synthase and glutamine synthetase enzymes, in plants treated with PH, where analogous results were shown by Colla et al. [4] for lettuce plants as well. Clearly, key amino acids and especially soluble peptides play numerous roles in plant physiology: (i) asparagine and glutamine are a bridge between the carbon and nitrogen cycle, (ii) glycine inhibits C3 plants photorespiration and ethylene is formed from methionine, and they form amides by detoxifying the cell from ammonia [19]. Noteworthy is the role of amino acids as natural chelators or iron carriers; they are involved in the induction of Fe III chelate reductase activity of tomato seedlings roots and leaves, an enzyme responsible for iron absorption [20].

In our study, there were no differences in yield and biomass between the biostimulant treatments used individually or combined. These results may highlight that the two tested biostimulants act on the wall rocket plant in the same way, and consequently did not produce a plant growth effect superior to the individual treatments. Furthermore, taking into account that neither the total nitrogen nor the nitrate content varied with the use of biostimulants, it suggests that there has been no ex novo absorption of nitrogen compounds from the environment to increase growth, but probably, biostimulants have induced the plant to mobilize and use its own nitrogen deposits already present. Ertani et al. [21] showed that two biostimulants, based on PH (one of vegetal origin and another of animal origin) added in different concentrations to the nutrient solution of maize seedlings, have a gibberellin like activity, which leads to an increment in the epicotyl length and an increment in the root dry weight of the seedlings. Considering the amount of N contained in the two biostimulants, the authors excluded their direct role as nutritional products, and their action was rather related to the activity that they can exercise as signal molecules and growth regulators [21]. The same authors showed a reduction in nitrate concentration in the roots and leaves of the treated plants, as compared to the control, an increase in the activity of nitrate reductase and glutamine synthetase, which is partially in line with our results where nitrate content in the third harvest in PH + PE treatment was lower than the control albeit statistically not significant. As in other Brassicaceae, rocket plants accumulate nitrate, because their leaves have an inefficient reductive system [22,23,24,25,26]. Nitrate is the major source of N available to vegetables [27,28,29,30], but it is harmful to human body because its reaction products can cause gastric cancer and other diseases [27,31]. *D. tenuifolia* leaves accumulate nitrate reaching up to 10 g kg^−1^ fw [32,33]. For this reason, the maximum levels of nitrate content allowed for rocket leaves, established from European Commission, are 7000 mg kg^−1^ fw (harvested from 1 October to 31 March) and 6000 mg kg^−1^ fw (harvested from 1 April to 30 September). In our work, nitrate content in rocket leaves did not exceed these limits (from 4557.8 to 4771.9 mg kg^−1^ fw). Interestingly, the foliar applications of vegetal-based biostimulants did not significantly modify the nitrate value compared to the control.

Rocket contains a high supply of potassium (4.7 g kg^−1^ fw) and calcium (3.1 g kg^−1^ fw) [16]. In humans, potassium is imperative for lowering blood pressure and hypertension, while calcium and magnesium reduce osteoporosis [34]. Furthermore, magnesium is a cofactor of kinases, H ^+^ -ATPase, polymerases and transaminases. It is involved in the light activation of Calvin cycle enzymes, and it is part of the chlorophyll porphyrinic ring [35,36]. Unlike nitrogen, phosphorus and sodium, potassium, calcium and magnesium contents were influenced by foliar applications of biostimulants. In particular, potassium content increased through the use of the two biostimulants, compared to the control, but without differences between treatments. In the second harvest, calcium content increased compared to the control, when the PH biostimulant was applied. Calcium increased in the presence of PE, and PH + PE, but was not significant between the last two treatments. In the third harvest, the increase in calcium compared to the control was evident with the use of PH and with the combined use of the two biostimulants, without significant differences between them. PH and PE application, increased calcium content, compared to control, in rocket plants analyzed by Caruso et al. [10], too. Similar results for magnesium and potassium were shown by Carillo et al. [37], in jute plants treated with the same PE. The presence of bioactive compounds in Trainer^®^ and Auxym^®^ (glutamic and aspartic acid that stimulate the metabolism of C and N and root-promoting peptides, auxins and cytokinins) may have increased the absorption of these nutrients, through an increase either in the root system, or in the transporters on radical cells membranes [38].

In our work, the trend of chlorophyll content was similar to magnesium, which makes sense since magnesium is an integral part of the chlorophyll molecule. Chlorophyll content was consistent with SPAD index results, which were significantly influenced by the biostimulants in the third harvest. SPAD index increased by 21.8% due to biostimulants treatment compared to the control, which led to a valuable doubling of the yield. This increase in yield is explained by a better plant photosynthetic efficiency reflected by the higher SPAD index values [17]. An increase in SPAD index due to PE and PH was also highlighted by Di Mola et al. [17] and Caruso et al. [10] on rocket.

Compounds such as phenols, carotenoids, flavonoids and vitamin C, represent the defensive power both in plants and humans, because they have the ability to block free radicals, which are harmful to proteins, lipids and DNA in the cells. They have been shown to be beneficial for treating a variety of diseases and illnesses (i.e., diabetes, inflammation and some types of cancer) [34,39]. Rocket plants are a good source of total phenolics (2.9 g kg^−1^ fw), total carotenoids (129 mg kg ^−1^ fw), and ascorbic acid (90 mg Kg^−1^ fw) [11,13,40]. Therefore, rocket consumption within the diet is recommended for maintaining human health [41,42], especially because it is richer than green lettuce, spinach and basil in carotenoids (44.4, 56.3 and 31.42 mg kg^−1^ fw, respectively; [43]) and in total phenols (1.3 g kg^−1^ fw [34], 0.87 g kg^−1^ fw [44] and 0.23 g kg^−1^ fw [45], respectively). As for vitamin C, rocket has a similar content to green lettuce (92 g kg^−1^ fw) but lower content than spinach and basil (281 and 180 g kg^−1^ fw, respectively; [43])

In our study, total ascorbic acid content (37.0 and 36.3 mg g^−1^ fw) did not show variation when the two biostimulants were applied individually, but their concentration increased when both biostimulants were combined together, without notable difference between the two harvests. This appears to be a synergistic effect of the two biostimulant treatments. The presumed mechanisms behind the premium quality wall rocket trait (high total ascorbic acid content), could be due to the activity stimulation of key enzymes involved in antioxidant homeostasis in cells (direct mode of action) [46]. Another putative indirect mechanism behind the synthesis and accumulation of total ascorbic acid could involve the increased nutrient assimilation (macro- and micronutrients) of biostimulant-treated plants, which could contribute to the synthesis of amino acids, tyrosine and phenylalanine [14]. The increase of ascorbic acid, following foliar treatment with PH and PE (from red grape and alfalfa plants), was also shown in *Capsicum cheinense* L. plants by Ertani et al. [46]. An increase of 30% in ascorbic acid content was linked to the overexpression of the L-galactono−1,4-lactone dehydrogenase enzyme (L-GalLDH) in lettuce plants [47]. This enzyme has an N-terminal target sequence rich in Ala, Leu, Arg and Ser and to a lesser extent Asp, Glu, Ile, and Val. In our work, the two biostimulants combination probably increased the amount of amino acids, which led to an overexpression of L-GalDH. The combination of PH and PE may represent a promising tool for increasing the nutritive value of perennial wall rocket.

## 4. Materials and Methods

### 4.1. Experimental Site, Plant Material and Greenhouse Conditions

The trial was conducted at the Department of Agriculture, University of Naples Federico II, Naples, Italy. *Diplotaxis tenuifolia* L. DC. cv. Nature was grown in an unheated greenhouse covered with a polyethylene film. The greenhouse dimensions were 15.0 × 30.0 × 2.0 m (width × length × height). Soil texture was sandy-loam, made of 76%, 17% and 7% of sand, silt and clay, respectively, and an organic matter content of 2.25% (w/w). Total nitrogen, P and exchangeable K, were 0.14 %, 32.8 mg kg^−1^ and 1372 mg kg^−1^, respectively. The soil electrical conductivity and pH were 512 mS·cm^−1^ and 6.9, respectively. The growing period lasted from November 2018 till April 2019. During the growing period the minimum and maximum air temperature ranged between 6 and 23 °C, whereas the relative humidity ranged between 40% and 60%. The plant density was 14.3 rosettes per square meter.

Three harvests were carried out based on standard practice of leaf maturation, harvesting the plants of the central rows and excluding those on the edge, at a length ranging from 12 cm to 15 cm. The cut was made at 3–5 cm above ground level, to ensure an adequate regrowth of the vegetative apex.

### 4.2. Experimental Protocol and Biostimulant Treatments

Three biostimulant treatments plus an untreated control were used: legume-derived protein hydrolysates (PH), tropical plant extract (PE), PH + PE and untreated control. The experimental protocol was carried out in a randomized complete blocks design with three repetitions, accounting for a total of 12 experimental units.

The PH Trainer^®^ (Italpollina SpA, Rivoli Veronese) is made of 75% free amino acids & peptides, 22% carbohydrates and 3% mineral nutrients [10]. While PE Auxym^®^ (Italpollina SpA, Rivoli Veronese) is made of 54% free amino acids and peptides, 23% mineral nutrients, 17% carbohydrates, 6% vitamins and 0.22% phytohormones [10].

The biostimulant treatments were carried out by foliar application on a weekly basis, starting when leaves reached 6 cm in length, to facilitate the applied substances absorption. PH was administered at a dose of 4 mL per liter, PE at a dose of 1 mL per liter and PH + PE at a dose of 2.0 + 0.5 mL per liter, respectively. The first treatments were applied on 7 December 2018, 35 days after transplanting, and were repeated every seven days until 3 January 2019. The first harvest was performed on 10 January 2019, 69 days after transplanting, thus marking the start of the new production cycle. On 1 February 2019, weekly treatments resumed, until 1 March 2019 with a second harvest on 8 March 2019. Weekly foliar treatments started on 15 March and continued until March 29, with the last harvest carried out on 4 April 2019. In regard to the crop management, manual weeding was carried out during the production cycle to avoid competition and irrigation was supplied by drippers. Each crop cycle received a fertigation dose of N 112 kg ha^−1^, P_2_ O_5_ 30 kg ha^−1^ and K_2_ O 90 kg ha^−1^ divided in three applications, where the first one was after transplant (first cycle) or straight after harvest (second and third cycle), and the last one was one week distant from harvesting. Noting that an organic fertilization prior to the start of the experiment was applied at a rate of N 38 kg ha^−1^, 10 P_2_ O_5_ kg ha^−1^ and K_2_ O 30 kg ha^−1^.

### 4.3. Harvest and Yield Determination

At each harvest, the yield of each plot was determined and expressed as g m^−2^ fw, considering only the plants of the central rows and excluding those around the edge of the plot for the purpose of evaluating the marketable yield. Subsequently, 200 g samples were prepared and put in an oven at 70 °C, until reaching a steady weight to determine dry weight (dw) through an analytical balance (XT120 A; Precisa Gravimetrics, Dietikon, Switzerland). Dry samples were ground and used for determining total nitrogen, nitrate and minerals. Fresh material from each treatment was divided in two portions and stored at −80 °C, where one portion was used to determine total ascorbic acid and total chlorophyll content and the other portion was subsequently freeze-dried for total phenols determination.

### 4.4. SPAD and Leaf Color Parameters

The soil plant analysis development (SPAD) index was evaluated on 30 entirely expanded leaves per plot, by using a portable Konica Minolta chlorophyll meter (model SPAD−502, Tokyo, Japan). While, on the upper surface of ten leaves per plot, a Minolta Chroma meter, CM−2600d (Minolta Camera Co. Ltd., Osaka, Japan) was used for determining the CIELAB color space parameters.

### 4.5. Total Nitrogen and Minerals Content

Total nitrogen was carried out following the Kjeldahl method [48]. For determining nitrate and other minerals content, 250 mg of dry material was used, based on the method described by Rouphael et al. [14]. An aliquot of each aqueous extract was analyzed with ion chromatography (ICS−3000, Dionex, Sunnyvale, CA, USA).

### 4.6. Total Chlorophyll Content

Based on the Lichtenthaler and Buschmann [49] method, 500 mg of fresh rocket leaves from each treatment was extracted in acetone (80%) for total chlorophyll content determination. The extract was kept in the dark for 15 min, then centrifuged at 3000 g for 5 min, and the supernatant was transferred to a cell for spectrophotometer measurements (Hach DR 2000, Hach Co., Loveland, CO, USA). 647 and 664 nm were the wavelengths used for chlorophyll a and b, respectively, and their sum served to obtain the total chlorophyll content.

### 4.7. Total Ascorbic Acid and Total Phenols

Total ascorbic acid and total phenols were assessed by spectrophotometric detection on fresh and dry plant tissues, respectively. Total ascorbic acid content was assessed based on the method proposed by Kampfenkel et al. [50], where the complex is quantified at an absorbance of 525 nm using calibration standard curve of ascorbic acid (Sigma Aldrich Inc., St. Louis, MO, USA). While total phenols content was determined by the Folin–Ciocalteau procedure [51], where the absorbance of the solution was read at 765 nm using calibration standard curve of gallic acid (Sigma Aldrich Inc., St. Louis, MO, USA).

### 4.8. Partial Budget Analysis

PBA was assessed to evaluate the net economic benefits that may accrue to the rocket farmers applying the plant-based biostimulants. The economic procedure adopted in the current greenhouse experiment has been described previously by Colla et al. [3]. For the three biostimulant combinations, the added costs and gross returns by applying the biostimulants compared to the untreated control treatment were calculated. In order to determine the added net return incurred by either PE or PH and combined plant-based biostimulants (PH + PE), the following formula was used: added net return = added gross return – added variable costs.

### 4.9. Statistical Analysis

The Shapiro–Wilk and Kolmogorov–Smirnov procedures were implemented to verify the data normal distribution. Then, the data were processed by the analysis of variance (One-way ANOVA) using the IBM SPSS 20 software package. The means of the four biostimulant combinations were confronted using Duncan’s test operated at *p* ≤ 0.05. For the determination of the interrelationship across the yield and rocket qualitative traits with reference to the biostimulant treatments, a principal component analysis (PCA) was applied implementing the suitable function PCA from the same SPSS software.

## 5. Conclusions

Modern agriculture faces two important goals, which are reducing environmental impact and increasing the production for an ever-growing world population. Biostimulants have been shown to sustain both of these goals, as they are considered plant-growth regulators and they increase nutrient use efficiency. Our results showed that Auxym^®^ (PE) and Trainer^®^ (PH) application increased the total yield and total dry biomass of greenhouse-grown rocket plants, 48% and 37% on average, compared to untreated plants. Although growing biostimulant-treated wall rocket incurred higher production cost, the higher total yield increased with the use of plant based biostimulants (especially PH and PH + PE) resulting in net economic benefits for growers. The two biostimulants resulted in a higher mineral status of potassium, calcium and magnesium, as well as chlorophyll content in treated plants. Interestingly, a synergistic effect of the two biostimulants was seen in the case of total ascorbic acid content, as this qualitative parameter was increased. Our data confirmed that biostimulants are an eco-friendly tool for helping growers to augment their yield and economic benefit when simply adding minor quantities of the products to their agricultural practices. Mixing these two types of biostimulants resulted in a synergistic effect on the quality of the produce with a half dose of each stimulant applied, which was shown to improve plant performance in terms of growth and quality. Nonetheless, more in-depth studies of biostimulant combination or maybe their alternated use is noteworthy for future studies on leafy greens.

## Figures and Tables

**Table 1:** Effect of foliar applications of protein hydrolysates (PH) and tropical plant extract (PE) alone or in combination on marketable yield and dry biomass of grenhouse cultivated perennial wall rocket.

Biostimulant Treatments	Yield (g m^−2^)			Total Yield (g m^−2^)	Dry Biomass (g m^−2^)			Total Dry Biomass (g m^−2^)
	Harvest I	Harvest II	Harvest III		Harvest I	Harvest II	Harvest III	
Control	462.8 ± 30.7	832.7 ± 15 b	403.7 ± 36 b	1699.1 ± 20 b	55.6 ± 1.5	67.3 ± 1.5 b	30.0 ± 2.9 b	153.0 ± 4.5 b
PH	493.0 ± 47.6	1246.6 ± 109 a	821.4 ± 111 a	2561.1 ± 174 a	53.6 ± 4.9	102.4 ± 13.5 a	61.1 ± 8.5 a	217.0 ± 16.5 a
PE	476.8 ± 59.8	1252.2 ± 27 a	726.8 ± 73 a	2455.7 ± 87 a	49.0 ± 4.8	99.1 ± 3.5 a	50.9 ± 3.0 a	198.9 ± 3.1 a
PH + PE	542.7 ± 63.9	1176.4 ± 111 a	816.8 ± 53 a	2535.9 ± 19 a	57.3 ± 5.8	95.1 ± 14.2 a	61.2 ± 5.5 a	213.5 ± 10.3 a
Significance	ns	*	*	***	ns	*	*	**

Notes*: Nonsignificant or significant at *p* ≤ 0.05, 0.01, and 0.001, respectively. Different letters indicate significant differences according to Duncan’s test (*p* = 0.05). All data are expressed as mean ± standard error, *n* = 3.

**Table 2:** Effect of foliar applications of protein hydrolysates (PH) and tropical plant extract (PE) alone or in combination on soil plant analysis development (SPAD) index and colorimetric parameters of greenhouse cultivated perennial wall rocket.

Biostimulant Treatments	Harvest II				Harvest III			
	SPAD	L*	a*	b*	SPAD	L*	a*	b*
Control	34.3 ± 1.20 b	39.7 ± 0.26	13.8 ± 0.34	19.8 ± 0.48	32.0 ± 1.53 b	40.4 ± 0.15	13.6 ± 0.39	19.6 ± 0.64
PH	38.7 ± 0.67 a	38.3 ± 0.57	13.3 ± 0.26	19.4 ± 0.43	37.7 ± 1.45 a	39.3 ± 0.77	14.1 ± 0.28	20.9 ± 0.64
PE	37.7 ± 0.88 a	39.0 ± 0.85	13.7 ± 0.52	20.0 ± 0.73	39.7 ± 1.45 a	39.8 ± 0.49	14.5 ± 0.14	21.3 ± 0.19
PH + PE	38.7 ± 1.76 a	39.1 ± 0.59	13.6 ± 0.34	19.5 ± 0.79	39.7 ± 1.33 a	39.5 ± 0.19	14.0 ± 0.31	20.3 ± 0.63
Significance	*	ns	ns	ns	*	ns	ns	ns

Notes*: Nonsignificant or significant at *p* ≤ 0.05, respectively. Different letters indicate significant differences according to Duncan’s test (*p* = 0.05). All data are expressed as mean ± standard error, *n* = 3.

**Table 3:** Effect of foliar applications of protein hydrolysates (PH) and tropical plant extract (PE) alone or in combination on leaf mineral composition of greenhouse cultivated perennial wall rocket.

Biostimulant Treatments	N (g kg^−1^ dw)		P (g kg^−1^ dw)		K (g kg^−1^ dw)		Ca (g kg^−1^ dw)		Mg (g kg^−1^ dw)		Na (g kg^−1^ dw)	
	Harvest II	Harvest III	Harvest II	Harvest III	Harvest II	Harvest III	Harvest II	Harvest III	Harvest II	Harvest III	Harvest II	Harvest III
Control	4.85 ± 0.10	5.03 ± 0.10	2.83 ± 0.19	2.93 ± 0.16	44.5 ± 0.51 b	37.5 ± 1.43 b	18.1 ± 0.10 b	26.2 ± 0.20 b	3.03 ± 0.24	2.90 ± 0.21 b	2.96 ± 0.10	2.64 ± 0.78
PH	5.01 ± 0.14	4.99 ± 0.18	3.20 ± 0.05	3.02 ± 0.14	48.1 ± 0.64 b	54.3 ± 1.34 a	23.8 ± 0.38 a	34.0 ± 0.54 a	3.13 ± 0.12	4.16 ± 0.35 a	2.16 ± 0.43	4.03 ± 0.14
PE	4.95 ± 0.18	4.71 ± 0.16	3.14 ± 0.13	3.08 ± 0.06	52.9 ± 2.26 a	53.9 ± 2.19 a	25.0 ± 0.56 a	30.0 ± 2.10 ab	3.31 ± 0.34	3.87 ± 0.24 a	2.57 ± 0.66	3.47 ± 0.67
PH + PE	4.89 ± 0.16	4.80 ± 0.15	2.87 ± 0.10	3.10 ± 0.17	48.3 ± 1.28 b	56.6 ± 1.61 a	25.1 ± 0.34 a	32.4 ± 1.47 a	3.32 ± 0.32	4.02 ± 0.10 a	2.02 ± 0.87	4.42 ± 0.22
Significance	ns	ns	ns	ns	*	***	***	*	ns	*	ns	ns

Notes*: Non-significant or significant at *p* ≤ 0.05, and 0.001, respectively. Different letters indicate significant differences according to Duncan’s test (*p* = 0.05), dw: dry weight. All data are expressed as mean ± standard error, *n* = 3.

**Table 4:** Effect of foliar applications of protein hydrolysates (PH) and tropical plant extract (PE) alone or in combination on qualitative parameters of greenhouse cultivated perennial wall rocket.

Biostimulant Treatments	Chlorophyll		Nitrate		Total Phenols		Total Ascorbic Acid	
	(mg g^−1^ fw)		(mg kg^−1^ fw)		(mg gallic acid eq. g^−1^ dw)		(mg g^−1^ fw)	
	Harvest II	Harvest III	Harvest II	Harvest III	Harvest II	Harvest III	Harvest II	Harvest III
Control	0.96 ± 0.14	0.88 ± 0.09 b	4630 ± 644	4428 ± 446	3.98 ± 0.07	4.71 ± 0.11	17.3 ± 3.22 c	14.9 ± 2.11 b
PH	1.34 ± 0.17	1.03 ± 0.06 ab	4558 ± 375	5611 ± 377	3.95 ± 0.38	5.03 ± 0.67	23.6 ± 1.11 bc	22.0 ± 2.08 b
PE	1.22 ± 0.14	1.11 ± 0.05 a	4634 ± 211	5897 ± 489	3.46 ± 0.20	4.32 ± 0.14	28.0 ± 0.53 b	31.3 ± 2.29 a
PH + PE	1.43 ± 0.18	1.22 ± 0.04 a	4772 ± 396	4380 ± 631	3.84 ± 0.06	4.46 ± 0.10	37.0 ± 1.79 a	36.3 ± 3.25 a
Significance	ns	*	ns	ns	ns	ns	***	***

Notes*: Nonsignificant or significant at *p* ≤ 0.05, 0.01 and 0.001, respectively. Different letters indicate significant differences according to Duncan’s multiple-range test (*p* = 0.05), dw: dry weight, fw: fresh weight. All data are expressed as mean ± standard error, *n* = 3.

**Table 5:** Added returns incurred by biostimulant applications under greenhouse conditions compared to untreated control.

Biostimulant	Added Gross Returns (€ ha^−1^)	Added Variable Costs (€ ha^−1^)				Added Net Returns (€ ha^−1^)
		Biostimulant treatment	Foliar spraying	Machine harvest	Total	
Protein Hydrolysates (PH)	11354.4	676.0	390	343.3	1409.3	9945.1
Tropical Plant Extract (PE)	9905.8	806.0	390	301.7	1497.7	8408.1
PH + PE	11276.6	741.0	390	333.4	1464.4	9812.2

The rocket selling (shipping point) prices of 1800 and 1300 € t^−1^ were used in calculating the gross returns of biostimulant-treated rocket production in first and second/third harvest, respectively; costs of biostimulants were provided by suppliers (PE: Auxym^®^ = 62.0 €/L−1; PH: Trainer^®^ = 13 €/L−1); costs of foliar spraying were calculated based on the information provided by local agricultural contractors; costs of machine harvest were calculated considering a machine with a harvesting capacity of 2 t h−1, a tractor with cart and six workers (two on top of the harvesting machine, one tractor driver and three in the cart for leaf box handling).

## Supplementary Materials

The following are available online at https://www.mdpi.com/2223-7747/9/7/922/s1, Table S1: Relative and cumulative percentage of total variance, eigen values and correlation coefficients of all agronomical and qualitative parameters of perennial wall rocket harvested three times during the cultivation cycle (HRV1, HRV2 and HRV3) with respect to the two principal components (PC1 and PC2).
