# Supplementary material for: Stand-Alone and Combinatorial Effects of Plant-based Biostimulants on the Production and Leaf Quality of Perennial Wall Rocket
Source: Plants (Basel). 2020 Jul 21;9(7):922. doi: 10.3390/plants9070922 (PMC7411840; doi:10.3390/plants9070922)
Supplement: Supplementary file 1 [file plants-09-00922-s001.pdf]

Table S1. Relative and cumulative percentage of total variance, eigen values and correlation coefficients of all agronomical and qualitative parameters of perennial wall rocket harvested three times during the cultivation cycle (HRV1, HRV2 and HRV3) with respect to the two principal components (PC1 and PC2).

Principal components	PC1	PC2	PC3
Relative variance (%)	66.2	20.8	13.0
Cumulative variance (%)	66.1	87.0	100.0
Eigen value	17.2	5.4	3.3
Eigen vectors			
SPAD HRV1	**0.995**	0.064	-0.071
Ca HRV2	**0.993**	0.038	-0.110
K HRV3	**0.974**	0.225	0.008
Total Yield	**0.973**	0.209	0.094
Mg HRV3	**0.960**	0.258	0.110
Yield HRV3	**0.956**	0.203	0.209
Na HRV3	**0.950**	-0.313	0.012
SPAD HRV2	**0.948**	0.186	0.258
Yield HRV2	**0.946**	0.303	-0.120
Chlorophyll HRV2	**0.941**	0.012	0.337
P HRV3	**0.889**	-0.316	0.333
TAA HRV3	**0.880**	-0.474	-0.043
Na HRV2	**-0.876**	-0.465	-0.126
Chlorophyll HRV3	**0.873**	-0.484	0.060
TAA HRV2	**0.844**	-0.501	0.193
Ca HRV3	**0.819**	0.391	0.419
K HRV2	**0.779**	0.071	**-0.624**
N HRV3	**-0.752**	0.440	0.491
Mg HRV2	**0.696**	**-0.635**	-0.336
Yield HRV1	**0.682**	-0.408	**0.607**
Nitrate HRV2	**0.303**	**-0.916**	0.263
N HRV2	**0.619**	**0.782**	-0.078
P HRV2	**0.603**	**0.771**	-0.206
Phenols HRV3	-0.291	**0.715**	**0.635**
Nitrate HRV3	0.435	**0.663**	**-0.609**
Phenols HRV2	-0.452	0.189	**0.872**

Boldface factor loadings indicate the most relevant characters for each principal component (PC1, PC2, PC3)
